# Metatranscriptomics reveals the molecular mechanism of large granule formation in granular anammox reactor

**DOI:** 10.1038/srep28327

**Published:** 2016-06-20

**Authors:** Samik Bagchi, Regina Lamendella, Steven Strutt, Mark C. M. Van Loosdrecht, Pascal E. Saikaly

**Affiliations:** 1 King Abdullah University of Science and Technology, Biological and Environmental Sciences and Engineering Division, Water Desalination and Reuse Research Center, Thuwal 23955-6900, Saudi Arabia; 2 Juniata College, Biology Department, Huntingdon, PA 16652, USA; 3 Delft University of Technology, Environmental Biotechnology, Department of Biotechnology, Van der Maasweg 9, 2629 HZ Delft, The Netherlands

## Abstract

Granules enriched with anammox bacteria are essential in enhancing the treatment of ammonia-rich wastewater, but little is known about how anammox bacteria grow and multiply inside granules. Here, we combined metatranscriptomics, quantitative PCR and 16S rRNA gene sequencing to study the changes in community composition, metabolic gene content and gene expression in a granular anammox reactor with the objective of understanding the molecular mechanism of anammox growth and multiplication that led to formation of large granules. Size distribution analysis revealed the spatial distribution of granules in which large granules having higher abundance of anammox bacteria (genus *Brocadia*) dominated the bottom biomass. Metatranscriptomics analysis detected all the essential transcripts for anammox metabolism. During the later stage of reactor operation, higher expression of ammonia and nitrite transport proteins and key metabolic enzymes mainly in the bottom large granules facilitated anammox bacteria activity. The high activity resulted in higher growth and multiplication of anammox bacteria and expanded the size of the granules. This conceptual model for large granule formation proposed here may assist in the future design of anammox processes for mainstream wastewater treatment.

The anaerobic ammonia oxidation (anammox) process is a promising alternative to conventional nitrogen removal from wastewater due to cost-effectiveness. Furthermore, it plays a significant role in the global nitrogen cycle where deeply branched members of the phylum *Planctomycetes* oxidize ammonia under anaerobic conditions using nitrite as the electron acceptor^1^. To date, six “*Candidatus*” anammox genera have been tentatively proposed and described based on enrichment cultures^2^,^3^. The first metagenome of enriched anammox “*Candidatus Kuenenia stuttgartiensis*” was released in 2006^1^, and the core step in anammox metabolism was unraveled. It included the reduction of nitrite to nitric oxide (NO) by cd_1_ nitrite reductase (NirS), condensation of ammonium and NO into hydrazine by hydrazine synthase (HZS) and oxidation of hydrazine to dinitrogen gas by hydroxylamine oxidoreductase (HAO)-like hydrazine dehydrogenase (HDH)^4^. Recently, the metagenome sequences of three more anammox species were reported: a marine species, “*Candidatus Scalindua profunda*”^5^, two wastewater species, “*Candidatus Brocadia fulgida*”^6^ and “*Candidatus Jettenia asiatica*”^7^, and all of them were different from the *K. stuttgartiensis* metagemone. For example, *Jettenia* and *Brocadia* employ a copper-containing NirK type nitrite reductase, instead of NirS, for reduction of nitrite to NO^6^,^7^. Metagenomic-based studies on these species unraveled the complexity and differences in metabolic pathways among different members of this fascinating group of bacteria.

While metagenomic approaches provide information on gene content, these technologies do not describe the activities of the microbial community and how these activities vary with respect to space, time, environmental factors or biotic interactions. Metatranscriptomics, or the large-scale sequencing of mRNAs retrieved from microbial communities, can shed light on microbial activities and their regulation. Metatranscriptomics overcomes the targeted nature of quantitative PCR (qPCR) and microarrays^8^,^9^ to measure gene expression in anammox cultures. However, metatranscriptomics is yet to be applied widely to study the activity of anammox communities due to several technical challenges, including the low relative abundance of mRNAs (1–5%)^10^, the difficulty of isolating prokaryotic mRNA due to the lack of poly(A) tails^11^, and the short half-life of mRNA^12^. Recent advances in mRNA enrichment from environmental samples^13^ and the reliability of commercially available kits for rRNA subtraction^10^ have facilitated the use of this technique to track low-frequency changes in gene expression of bacterial communities associated with complex microbial communities such as those involved in the granular anammox process.

The anammox process has been implemented for treatment of ammonia-rich side-stream wastewater as granular aggregates in which anammox bacteria grow at the core surrounded by ammonia-oxidizing bacteria (AOB) and heterotrophs^14^. Current technological development has been focused on applying the granular anammox process to main-stream sewage under low-temperature conditions^15^,^16^. Performance under low temperature can deteriorate due to growth of nitrite-oxidizing bacteria (NOB), which is a competitor for anammox bacteria. Previous research reported spatial segregation of granules in granular anammox reactor where large granules (greater than 500 μm) reside at the bottom of the reactor^16^. Large anammox granules can overcome competition from NOB by providing a niche for anammox growth^16^. Thus, large granules are beneficial for anammox activity. Also, physicochemical analysis revealed higher anammox activity in large granules^17^, but little is known about the molecular mechanism of anammox growth and multiplication that lead to the formation of large granules. Thus, from a technological perspective, it is important to know how anammox bacteria grow and multiply inside the granules and to understand the temporal and spatial (top vs. bottom) changes in gene expression in granular anammox sludge.

Here, we employed rRNA-subtracted metatranscriptomics to track changes in gene expression of bacterial communities involved in the granular anammox process over space and time in a lab-scale sequencing batch reactor (SBR). The main objectives of this study were to: (i) define the microbial community structure of anammox communities using qPCR and 16S rRNA gene sequencing; (ii) reconstitute the enzymatic machinery for key pathways in the anammox process; and (iii) measure their expression dynamics over space and time using metatranscriptomics. The application of rRNA-subtracted metatranscriptomics enabled in-depth bioinformatics analyses, which revealed a robust microbial community with an active role in treatment of ammonia-rich wastewater. Based on the results, we hypothesized the molecular mechanism of anammox growth and multiplication that led to the formation of large granules.

## Results and Discussion

### Enrichment of Anammox Bacteria

For enrichment of anammox bacteria, the laboratory-scale SBR was operated for a period of 79 days. A stable condition with average removal of 86 ± 3% of total nitrogen (TN) was realized within several days of reactor start-up (Supplementary Figure S1A). The effluent ammonia concentration was below the detection limit by day 73. The average nitrite to ammonia consumption ratio of 1.35 ± 0.19 was in line with anammox process stoichiometry (Supplementary Figure S1B).

**Figure 1:**
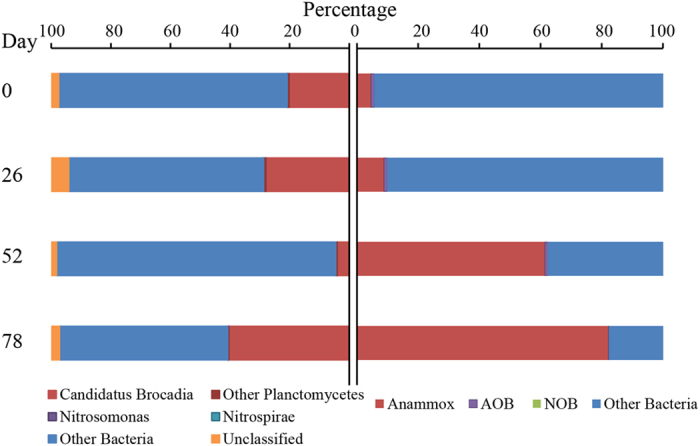
Comparison of the relative abundance of major N-cycle microorganisms per sampling day (average of top and bottom) based on 16S rRNA gene sequences (left) and qPCR (right). 16S rRNA gene sequences from Day 52 are included only for the top sample. ‘Other bacteria’ in 16S rRNA gene sequences included 30 different phyla and ‘unclassified’ are sequences not matched to any know organisms. For detailed information about the phyla abundance see Supplementary Figure S3.

Biomass was withdrawn from the reactor at different time periods to perform microbial analyses. As shown in Supplementary Figure S2, the biomass was reddish in appearance and the SEM observation of granules showed the presence of coccoid cells that are characteristic of anammox bacteria. We analyzed the population distribution by qPCR and 16S rRNA gene sequencing to understand the community composition and enrichment of anammox bacteria (Fig. 1). Estimation of total bacteria, anammox, AOB and NOB by qPCR showed a gradual increase in the anammox copy number from 9.7 log copies mL^−1^ to 13 log copies mL^−1^ over a period of time (Supplementary Figure S3A). A similar trend was observed by 16S rRNA gene sequences in which the relative abundance of anammox bacterial genus *Candidatus Brocardia* increased over time with the exception of the Day 52 samples (Fig. 1). The Day 52 bottom sample was an outlier as determined by PC-ORD analysis (See Supporting Information) and removed from further analysis. In general, 16S rRNA gene sequence data from the samples showed a more diverse population with a much smaller *Ca. Brocadia* and other *Planctomycetes* fraction as compared to qPCR data (Supplementary Figure S3B). In a similar comparative study using FISH and 16S rRNA gene sequencing on an anammox consortium, van de Vossenberg, Woebken^5^ showed that anammox bacteria were underrepresented in the sequence data retrieved from a bioreactor. The under-representation of anammox bacteria has been observed in other sequencing efforts and may be caused by incomplete DNA extraction, overrepresentation of GC-rich members of the community^6^, or variable per-genome copy number of 16S rRNA gene^18^.

Compared to the anammox bacterial population, AOB remained relatively stable throughout the experimental period (Supplementary Figure S3). Previous studies also observed similar stable AOB populations under anoxic conditions^8^,^19^. We also quantified both *Nitrospira*-like and *Nitrobacter*-like NOB in the reactor. The percentage of NOB compared to total bacteria was minimal (less than 0.001%) during the entire experimental period. Similarly, a low abundance of *Nitrospira* (less than 0.01%) was observed by 16S rRNA gene sequencing (Fig. 1). Overall, both qPCR and sequencing confirmed the enrichment of anammox bacteria belonging to the genus *Brocardia* in the reactor. Recent phylogenetic analysis of the seeding biomass also confirmed the presence of *Brocadia* as the dominant bacteria in the anammox granules^20^.

### Spatial distribution of Anammox granules

Although anammox bacteria were enriched in the reactor over a period of time, there were differences between the top and bottom samples in terms of size distribution, density of granules and community composition. Particle characterization showed that the number of granules (greater than 500 μm in size) at the bottom of the reactor increased over time (data not shown). Earlier research showed segregation of anammox granules in the settled sludge bed and accumulation of large granules at the bottom due to small differences in settling velocity^16^,^17^,^21^. The other parameter that distinguished top and bottom samples was density, which was always higher for the bottom biomass than for the top biomass (Supplementary Figure S4). Thus, density played an important role in the segregation of the biomass. This is in contradiction to Winkler, Kleerebezem^21^ who suggested that mainly granular diameter and not density caused segregation of the biomass.

Winkler *et al*.^16^ reported spatial segregations in granular anammox reactor with large granules residing at the bottom of the reactor. Also, they reported that the bottom granules were dominated by anammox and AOB while NOB appeared in the top layer. In our study, qPCR revealed higher growth of anammox bacteria in the bottom biomass (Supplementary Figure S5). By day 79, anammox bacteria made up ~95% of the total bacterial population in the bottom biomass while ~70% of total bacteria in the top layer was composed of anammox bacteria. However, the percentage of NOB compared to total bacteria was minimal (less than 0.001%) throughout the experimental period. This was most likely a result of the anoxic conditions maintained in our reactor, where the open headspace served as the only source of oxygen diffusion. Overall, there was spatial distribution of granules in the reactor where the bottom was dominated by large granules having higher relative abundance of anammox bacteria.

### Metatranscriptome sequence analysis

To assess which nitrogen metabolism genes were expressed and differential expression in the top versus bottom biomass, we studied the metatranscriptomic profiles of anammox communities. A total of 17–24 million metatranscriptome sequence reads were generated from each sample, with a mean length of 101 bp (Table S1). *De novo* assembly of reads after removal of redundant sequences yielded 7,869 to 12,052 contigs. By BLASTn annotations to the nr database, 18–24% of the assembled contigs had predicted features for rRNA genes, which was relatively low compared to the rRNA content of prokaryotic cells (>80%) and most likely was a result of subtractive hybridization of rRNA transcripts. Previous research on enrichment of mRNA reads by subtractive hybridization reported similar percentages of rRNA sequences^11^,^12^,^22^. The assembled contigs were assigned to 3,201–5,374 COGs and 5,262–8106 KEGG genes in our samples (Table S1). Normalized expression of COG and KEGG functional categories showed that the relative abundance of transcripts assigned to COGs and KEGGs varied between top and bottom samples. Also, housekeeping genes were highly expressed across all samples (Supplementary Figures S6 and S7, respectively). For example, several of the most abundant COGs were from the ‘Translation, ribosomal structure and biogenesis’, ‘Intracellular trafficking and secretion’ and ‘Translation, ribosomal structure and biogenesis’ categories (Supplementary Figure S6). Ribosomal proteins, RNA polymerases, and chaperon proteins were among the highest expressed KEGG orthologs. Interestingly, a nitrate/nitrite transporter (COG 2223) and a nitrate reductase (K00370) were highly expressed across the samples, suggesting nitrogen-transforming processes were central in the sampled reactors (Supplementary Figure S7). Taxonomic annotation of nitrogen metabolism pathways in the KEGG website showed a wide variety of taxa representing genes for nitrogen metabolism (data not shown). Genes directly associated with anammox could not be identified from the COG or KEGG database as it does not contain annotated anammox genes; previous work also showed that KEGG was not able to classify genes associated with anammox from an enriched anammox culture^5^.

### Genes for Anammox Metabolism

Since both KEGG and COG databases failed to identify genes considered important for anammox process, metatranscriptome data were mapped to the *K. stuttgartiensis* genome (CT573071 through CT573074 and CT030148.2). Although mapping our expression data with the *Brocadia* genome would have been appropriate, no full-length sequence of the *Brocadia* genome was available in the database at the time of analysis. An average of ~6.2% reads mapped to the *K. stuttgartiensis* genome (Table S1), which is considerably higher than previous results in which samples were directly taken from an anammox bioreactor^5^. The following sections detail the expression of anammox genes and their implication for anammox metabolism.

### Ammonium, nitrite and nitrate transport

Anammox bacteria must acquire their substrates, ammonium and nitrite in particular, from the surrounding environment for catabolism. As such, anammox bacteria express several genes related to the major facilitator superfamily (MFS) to facilitate transport of major substrates. For example, anammox bacteria use the AmtB/Rh superfamily of proteins for ammonia uptake and FocA for nitrite/formate transport. In our metatranscriptomic analysis, we found expression of five *amtB* genes in our samples (Table 1) similar to *K. stuttgartiensis* which has five distinct *amtB* genes: Kustc1009, Kustc1012, Kustc1015, Kuste3690 and Kustc0381^4^,^23^. But unlike *K. stuttgartiensis*, where Kustc1009, Kustc1012, and Kustc1015 are located in same gene cluster, only two genes clustered (Kustc1009 and Kustc1012) in a single contig (1778_c0_g1_i1). These genes might be co-transcribed and translated separately after posttranscriptional modification. The other gene homologous to Kustc1015 was present in a completely separate contig (2418_c0_g1_i1) although both Kustc1012 and Kustc1015 have been predicted to mediate ammonium transport from the periplasm into the cytoplasm^23^. In the *Jettenia* metagenome, Kustc1015 is placed in a separate contig as well^24^.

In our metanscriptome, we identified four *focA* transcripts (kusta0004, kustd1720, kustd1721, and kuste4324), which facilitate the transport of nitrite across anammox membranes (Table 1). The four genes were present in tandem in two contigs (1888_c0_g1_i1 and 1390_c0_g1_i1) similar to tandem repeats found in the *K. stuttgartiensis* genome^23^. It is presumed that all four FocA proteins play a significant role in nitrite transport with one protein localizing on the anammoxosome while the counterpart localizes in the cytoplasmic membrane for transport of nitrite across the membranes^23^. For nitrate transport, two genes encoding high-affinity nitrate/H + symporters *narK1* (kuste2308 and kuste2335) were expressed in our samples. Both *narK1* transcripts were present in a single contig (1847_c0_g2_i1) and, similar to FocA proteins, they facilitate nitrate transport across membranes into the anammoxosome.

### Nitrite/nitrate conversion

After transport of substrates, most of catabolic reactions occur inside the anammoxosome. One of the most important reactions is the reduction of nitrite, providing NO for HZS. In the *K. stuttgartiensis* genome, cytochrome cd_1_-type nitrite reductase (NirS) catalyzes the reduction of nitrite to NO^4^. *NirS* has been highly expressed in the marine anammox organism, *S. profunda*^5^. However, the contig 2640_c0_g1_i1 in our transcriptome mapped poorly to *nirS* (kuste4136) of *K. stuttgartiensis* (Table 1). Only a small proportion of RNA fragments map to *nirS* (TPM 11.5) as compared to other anammox genes expressed in our dataset (Table 1). In *K. stuttgartiensis, nirS* was transcribed at a low mRNA level and barely detectable in the proteome^4^. It is possible that low amounts of a highly active NirS suffice as a nitrite reductase or that some additional nitrite reductase is present in the anammox community^23^. Indeed, the *nirS* gene was absent from *B. fulgida*^6^*, Ca. Jettenia caeni*^25^ (previously designated as KSU-1)^26^ and *J. asiatica*^7^, where an alternative copper-containing nitrite reductase, *nirK*, was detected as the major nitrite reductase. In our metatranscriptome, contig 1960_c0_g1_i1 had a higher coverage with the *nirK* gene in KEGG, but mapped to Kuste2479 in *K. stuttgartiensis.* Kuste2479 was categorized as hydroxylamine oxidoreductase (HAO) fused to a multicopper oxidase. Kartal *et al*.^23^ suggested HAO-like genes kustc0458 and kuste4574 as possible candidates for nitrite reduction in anammox bacteria and HAO homologues (2094_c0_g14_i8 and 2112_c3_g3_i6) were expressed in our samples. The results indicate the possibility that anammox may have multiple enzymes catalyzing the nitrite reduction, providing essential metabolic flexibility in response to environmental changes.

As observed during reactor operation, nitrate formation is a hallmark of anammox growth (Supplementary Figure S1). In anammox bacteria, the oxidation of nitrite to nitrate requires a dedicated nitrite:nitrate oxidoreductase (Nxr) system. In our contig assembly, there was a high abundance of *nar*G and *nar*H genes (Table 1). The narGH complex is the catalytic portion of the canonical nitrate reductase and is thought to catalyze nitrate reduction in nitrate-respiring species^22^,^27^,^28^, but in anammox bacteria, narGH functions in the reverse direction and catalyzes the oxidation of nitrite to nitrate. High sequence similarity of narGH to the Nxr component of the nitrite-oxidizing species ‘*Ca. Nitrospira defluvii*’ further confirms its role in nitrite oxidation^29^ However, the Nxr complex in anammox bacteria can also act as a true nitrite reductase when oxidizing small organic molecules with nitrite as an electron acceptor^30^.

### Hydrazine metabolism

One of the most intriguing properties of anammox bacteria is the condensation of ammonia with NO to form hydrazine. HZS, a heterotrimeric protein encoded by gene cluster kuste 2859–2861 (*hzsCBA*) in the *K. stuttgartiensis* genome^4^, catalyzes this reaction. The metatrancriptome data mapped well to all three sub-units of *hzs* genes (2587_c1_g1_i9, 2154_c1_g5_i8, and 2154_c1_g5_i5) with identities between 80–86% and were proportionally among the most abundant transcripts by TPM for gene involved in nitrogen metabolism. Previous studies reported high expression of *hzs* in transcriptome data from *K. stuttgartiensis*^4^. Thus, HZS is central to the anammox metabolism and expression of HZS in our samples is consistent with previous findings.

The final step in nitrogen conversion, the oxidation of hydrazine to N_2_, is catalyzed by an octaheme hydroxylamine oxidoreducatse-like (HAO-like) protein, hydrazine dehydrogenase (HDH). The *K. stuttgartiensis* genome codes for 10 different HAO-like HDH proteins, six of which are highly expressed at the transcriptional and protein levels^4^,^23^. Out of the 10 reported HAO-like HDH proteins, eight have been expressed in our samples (Table 1). Among the HAO-like HDH proteins, the kustd0694 and kustd1340 orthologs mapped to a single contig (1955_c1_g1_i2) in our samples, similar to the *J. asiatica* metagenome^7^. These two proteins have been shown to be physiologically important HDH proteins, as they catalyze the four-electron oxidation of hydrazine to N_2_^4^. The expression of other HAO-like HDH proteins suggests important physiological functions, but their detailed functions remain unknown^23^.

### Carbon fixation

Anammox bacteria live autotrophically by fixing atmospheric CO_2_ by the acetyl-CoA pathway. A complete acetyl-CoA pathway was identified and the function of a key enzyme, acetyl-CoA synthase (ACS), has been demonstrated in *K. stuttgartiensis*^1^. Two genes encoding the alpha and beta subunit (*acsA* and *acsB*) have been retrieved in our samples (2117_c0_g1_i2 and 1922_c0_g1_i4) having high identity to the genes within *K. stuttgartiensis* (Table 1). This indicates that the dominant anammox bacteria in our samples most likely use the same carbon fixation pathway as reported in other species.

Overall, our metatranscriptome analysis has enabled a holistic overview of the expression of the enzymatic machinery driving the anammox process. Since *Brocadia* was the dominant anammox bacteria in our reactor, the work presented here also highlights the difference in gene expression between *Brocadia* and *K. stuttgartiensis* and other anammox genera. One distinct difference is the expression of nitrite reductase gene. In *S. profunda and K. stuttgartiensis* genome assemblies, *nirS* genes have been expressed as nitrite reductase^4^,^5^. Both genera are k-strategist and maintain low diversity in natural environment^31^. On the other hand, *Brocadia* is r-strategist and overgrow in high ammonium and nitrite concentration^32^. The expression of HAO-like contigs (2094_c0_g14_i8 and 2112_c3_g3_i6) and *NirK* as the possible nitrite reductase provided the necessary flexibility and high growth for *Brocadia* in our reactor.

### Temporal and spatial (top vs. bottom) gene expression

**Figure 2:**
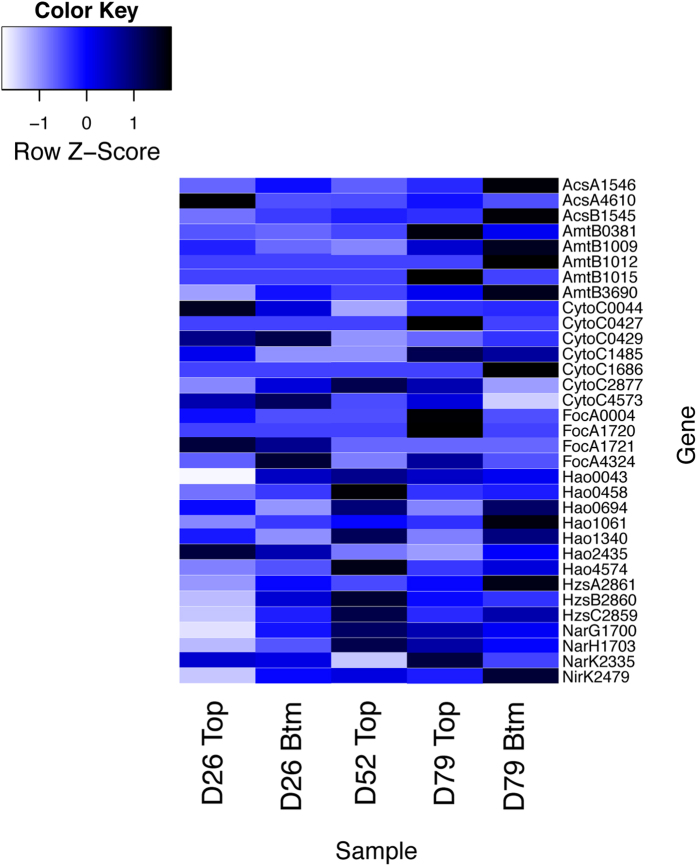
Temporal and spatial differences in nitrogen metabolism gene expression. Per-base coverage of raw reads mapped to select genes was normalized to gene length and number of total reads per sample. Genes were labeled with the locus tag number from *K. stuttgartiensis* to discriminate between homologs of similar proteins within the same genome. The colors of the heatmap are mapped linearly to the Z-scores, to highlight differences in individual genes, with lighter or darker blue representing lower or higher expression, respectively.

Although key anammox enzymatic machinery for anammox metabolism has been expressed in all samples, there were considerable differences in temporal and spatial gene expression within the reactor (Fig. 2). Based on normalized gene expression profiles, Day 79 samples had higher expression of MFS proteins like AmtB and FocA. As described earlier, these contigs contain gene clusters that play important roles in ammonia and nitrite transport. The higher expression of these genes during the later stage of reactor operation enhanced the transport of the substrates inside the anammox cells. Among the key catabolic enzymes, one subunit of hydrazine synthase (HzsA) and nitrite reductase (nirK) had higher expression in the Day 79 samples. Expression of both transport proteins and catabolic enzymes indicates higher activity inside anammox cells under favorable conditions. Most of the HAO-like HDH had high expression throughout the experimental period, once again verifying its role in removing highly toxic hydrazine from the cells. Both alpha and beta subunits of acetyl-CoA synthase (ACS) had higher expression in the Day 79 samples (Fig. 2). Recent research showed that anammox bacteria have a more versatile metabolism than previously assumed and ACS can oxidize organic carbon using nitrate as an electron acceptor^30^. Since exogenous organic carbon was not added to the reactor, it is tempting to assume that the source of organic carbon was due to endogenous decay of bacteria, and which triggered higher expression of ACS.

There were also considerable differences between top and bottom samples in terms of community composition and gene expression. Using qPCR, we observed higher abundance of anammox bacteria in the bottom samples (Supplementary Figure S5). Both top and bottom samples from Day 79 had higher expression than did the earlier samples (Fig. 2). One interesting difference between top and bottom samples from Day 79 was the higher expression of the nitrite transporter FocA in the top (Fig. 2). Given that we detected the presence of NOB during this period, we suggest that anammox expressed higher nitrite transporters to cope with NOB competition. Previous research showed lateral gene transfer between NOB and anammox^29^ and the possibility that NOB genes map to the *K. stuttgartiensis* genome cannot be discounted here. Overall, most of the key anammox metabolism genes had higher expression during the later stage of reactor operation with the bottom samples from Day 79 having the highest coverage.

### The molecular mechanism of large granule formation

**Figure 3:**
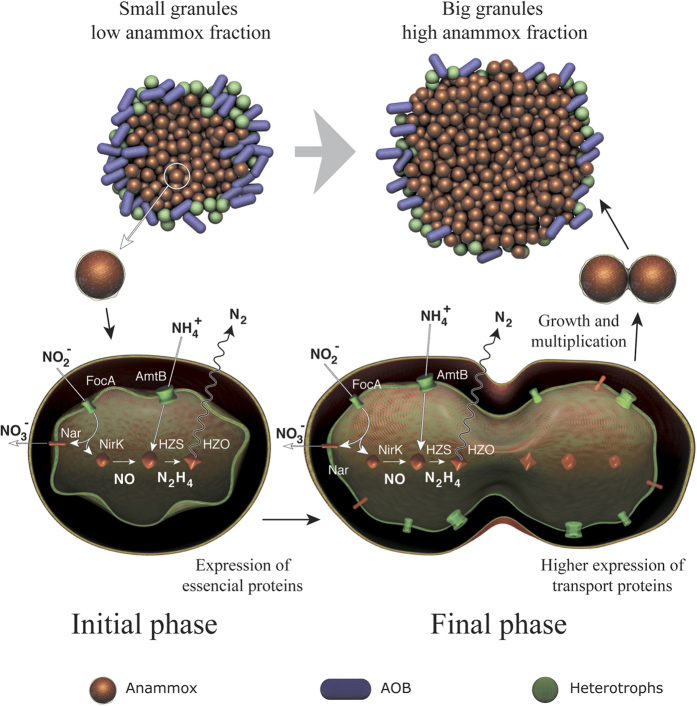
Overview of the proposed molecular mechanism of large granule formation. Anammox bacteria highly expressed transport proteins (AmtB and FocA) and catabolism enzymes (HZS and NirK) during the later stages of reactor operation. This led to growth and multiplication of anammox bacteria and increases in the anammox volume fraction in large granules. Figure 3 was created by Academic Writing Services, KAUST, Saudi Arabia.

The anammox process has been well established for treatment of ammonia-rich wastewater like anaerobic sludge, digester effluent, landfill leachate and other wastewater containing low organic load^33^. Such conventional anammox processes employ high temperature and/or low dissolved oxygen (DO) to selectively control NOB activity to achieve good removal efficiency^16^,^17^. Since selective inhibition of NOB under low-temperature conditions is difficult due to a similar growth rate of AOB and NOB, a new strategy of segregation of anammox granules to select specific microbial groups has been proposed^16^. Differential growth of anammox bacteria was observed in the reactor, most likely due to niche specialization by anammox bacteria in larger granules, while NOBs are favored to grow in smaller granules due to higher aerobic volumes^17^,^34^. Thus, large granules are better suited for treatment of wastewater under low-temperature conditions. For the first time this study provides the molecular basis for the observed increase in large granules over the enrichment period inside a granular anammox reactor (Fig. 3, Supplementary Animation 1).

Metatranscriptomics revealed higher expression of ammonia and nitrite transport proteins (AmtB and FocA, respectively) during the later stage of reactor operation (Fig. 2). Once anammox bacteria were enriched, the high expression of MFS proteins enhanced the transport of major substrates inside the anammox cells and increased their activity. Anammox bacteria increase their activity by expressing key catabolic enzymes like HZS and NirK, resulting in increased growth of anammox bacteria inside the granules and in the increase in granular size (Fig. 3). Both qPCR and 16S rRNA gene sequencing allowed us to observe an increase in anammox abundance during the later stage of reactor operation (Fig. 1). Thus, small granules expanded to large granules by growth of anammox bacteria inside the core of the granules during the enrichment phase due to the expression of key metabolism genes (see the Supplementary Animation 1). This confirms the hypothesis proposed by Volcke *et al*.^34^ that anaerobic granules increase size by growth of anammox bacteria inside the core of granules.

## Conclusion

In summary, anammox bacteria belonging to genus *Brocadia* was enriched in our reactor. By applying rRNA-subtracted metatranscriptomics, we were able to identify all essential genes for Brocadia metabolism based on comparison with other anammox genera. Additionally, by size distribution and qPCR we could observe the spatial segregation of anammox granules where large bottom granules provide a preferential niche for anammox bacteria. Based on expression dynamics over time, we observed higher expression of transport proteins (AtmB and FocA) and key metabolic enzymes (HzsA and NirK) during later stage of reactor operation, which facilitate higher activity, and growth of anammox bacteria at the core of granules. The growth and activity of anammox bacteria inside the core increased granular size. This insight into the large granule formation will assist in the future design of anammox processes for mainstream wastewater treatment.

## Methods

### Reactor operation

A SBR made of acrylic glass with 4-L working volume was operated for a period of 79 days. The reactor was inoculated with granular anammox sludge from a full-scale anammox reactor at Rotterdam Dokhaven, The Netherlands. Synthetic wastewater specified for anammox^35^ containing ammonia and nitrite at a 1.3 ratio was employed to establish the anammox community. Influent ammonia and nitrite concentrations were maintained at 92 ± 9 mg N L^−1^ and 128 ± 5 mg N L^−1^, respectively, until day 52 and then increased to 190 ± 12 mg N L^−1^ and 227 ± 15 mg N L^−1^, respectively. The reactor was operated in a sequencing fed batch mode (Supplementary Figure S8), with a 4 h cycle consisting of a 60-min anoxic feeding period, followed by a 150-min oxygen limited mixing phase, a 20-min settling period, and a 10-min effluent withdrawal. During effluent withdrawal, one-fourth of the reactor volume (1 L) was discharged. Mixing was provided a by magnetic stirrer at a rate of 50 rpm and recirculating a 5% CO_2_ + 95% Argon gas mixture which also eliminated dissolved oxygen from the reactor. Feeding, decanting, and sludge wastage were performed using a peristaltic pump (Masterflex L/S, Cole-Parmer Instrument Co., Chicago, IL) programmed by a timed controller (ChronTrol Corp., San Diego, CA). The reactor was operated at a fixed hydraulic retention time (HRT) of 16 hours. It was kept in a temperature-controlled room to maintain a constant temperature of 21 ± 1 °C. The pH was maintained at 7.0 ± 0.2. The performance of the SBR was monitored by measuring ammonia, nitrate, and nitrite concentrations on a daily basis except on weekends.

### Chemical analysis

Samples for chemical analysis were filtered with 0.45 μm syringe filters (Whatman, Maidstone, UK). Ammonia (NH_4_^+^-N), nitrate (NO_3_^−^-N) and nitrite (NO_2_^−^-N) were measured colorimetrically using the HACH Test-N Tube reagent kit (catalog numbers 26069-45, 26053-45 and TNT839, respectively) as described in the manufacturer’s protocol. Nitrogen removal (ΔN) was calculated as follows; ΔN = [(NH_4_^+^−N_inf_) + (NO_2_^−^−N_inf_)] – [(NH_4_^+^−N_eff_) + (NO_2_^−^−N_eff_) + (NO_3_^−^−N_eff_)].

### Biomass collection and DNA extraction

Biomass was collected from the top and bottom of the reactor on days 0, 26, 52 and 79. Fractions of samples (5 mL each) were fixed immediately in 2% (w/v) paraformaldehyde/phosphate buffer saline pH 7-2-7-4 (fixation buffer) for granule size characterization and scanning electron microscopy (SEM). Samples for DNA/RNA extraction were collected in triplicate (1 mL each) from the SBR during the reaction phase and centrifuged at 12,000 × g for 1 min and the supernatant was decanted. RNA samples were immediately re-suspended in RNAlater (Qiagen, Valencia, CA) and stored frozen at −80 °C until use. Genomic DNA was extracted from the freshly collected samples using the FastDNA® SPIN Kit (MP biomedicals, CA) according to the manufacturer’s protocol. The concentration of extracted DNA was measured with a Qubit® 2.0 Fluorometer (Invitrogen, Carlsbad, CA). All DNA samples were stored at −20 °C until used. Quantitative PCR (qPCR), sequencing and sequence analysis of 16 S rRNA genes is described in Supplementary Information.

### RNA extraction, rRNA subtraction and cDNA synthesis

Day 0 samples were not preserved in RNAlater and thus were not subjected to RNA extraction. All subsequent samples collected from the reactors were subjected to total RNA extraction using the RNeasyMini kit (Qiagen, Valencia, CA) according to the manufacturer’s instructions. Extracted RNA was treated with DNase (TURBO DNA-free kit, Ambion, Austin, TX) for removal of contaminant DNA and cleaned by an RNeasy MinElute Cleanup Kit (Qiagen, Valencia, CA) as per the manufacturer’s protocol. Purified RNA extracts were subjected to the MICROBExpress (Ambion) subtraction method as per the manufacturer’s instructions. Subtractive hybridization reduces bacterial rRNA transcript abundance, thus increasing enrichment of non-rRNA sequences^10^. Enriched mRNA was amplified using the MessageAmp II-Bacteria kit (Ambion) as described previously^36^. Briefly, enriched mRNA was polyadenylated using *Escherichia coli* poly(A) polymerase and converted to double-stranded complementary DNA (cDNA) via reverse transcription. The quantity and quality of total RNA, mRNA and cDNA were verified after each step using a Qubit® 2.0 Fluorometer (Invitrogen, Carlsbad, CA) and a 2100 Bioanalyzer RNA Nano 6000 assay (Agilent Technologies, Germany) as per the manufacturer’s instructions. The quantity of the Day 52 bottom (D52.btm) sample was low and was not included for further analysis. The double-stranded cDNA from other samples (five in total) were digested, purified, and sequenced by Illumina HiSeq (Roche, Indianapolis, IN) at the Bioscience Core Laboratory at KAUST. All transcript sequences from this study have been deposited at NCBI Sequence Read Archive (SRA) under project accession number 527639.

### Metatranscriptomics data analysis

Paired-end Illumina reads from each of the five samples were interleaved and checked for quality with FastQC (http://www.bioinformatics.babraham.ac.uk). Bases that were assigned a Phred score of greater than 20 were retained. Redundant sequences were removed using a normalization script packaged in Khmer^37^ with a k-size and coverage of 20, resulting in reducing the data down to approximately 10% of the initial reads. Paired reads were assembled into contigs using Trinity (v2.1.0)^38^. Taxonomic annotations were assigned to contigs using the BLASTn algorithm (v2.2.28+) against the nucleotide (nt) database (available from ftp://ftp.ncbi.nlm.nih.gov/blast/db/) with an E-value cutoff of less than 1e^−6^ and percentage identity greater than 90%^39^. Contigs were annotated against the Clusters of Orthologous Groups (COG) CDD database (v1.0) using the rpstblastn algorithm and the KEGG genes database using BLASTp, both at an E-value less than 1e^−6 ^40,42. A manually curated database of important genes in nitrogen metabolism was queried against all assembled contigs using tBLASTx. TPM (transcripts per million) was calculated using RSEM (RNA-Seq by Expectation Maximization)^43^.

To better compare samples, raw reads were submitted to MG-RAST for annotation^44^. Using the Hierarchical Classification feature of MG-RAST, KEGG Orthology (KO) annotations were generated using a minimum E-value of 1e^−6^, a minimum sequence identity of 60%, and a minimum alignment length of 15 bp. Abundances of resultant functional annotations were normalized to rpoB abundance^45^. As KEGG does not contain the gene for hydrazine synthase (*hzsA*), all nucleotide sequences matching *hzsA* were downloaded from NCBI and raw reads were searched against these *hzsA* sequences using BLASTn with an E-value cutoff of 1e^−6^. The number of hits was also normalized to rpoB abundance in each sample. Using rpoB-normalized values, relative abundance bars and heatmaps were generated in R using the gplots package^46^.

### Granule characterization by morphology

Granule morphology was determined using the Morphologi G3 particle characterization system (Malvern Instruments Limited, Malvern, UK). This automated particle imaging system was used to measure size, shape parameters and density of anammox granules. Density was measured as the intensity mean, which is the average of the pixel greyscale levels in the object. Particle imaging was conducted with a 2.5× lens with a resolution range between 13 and 1000 μm and analyzed according to a Standard Operating Procedure (SOP) with Morphologi 7.2 software (Malvern Instruments Limited, Malvern, UK). Images of samples with overlapped regions and multiple particles joined together were manually removed. Biomass samples were classified based on circle equivalent (CE) diameters as follows: 30–200 μm as floccules, 200–500 μm as small granules and greater than 500 μm as large granules^47^. Samples were analyzed by plotting a dendrogram and trend-plot based on the CE diameter (size) and intensity mean (density). The CE diameter is the diameter of a circle whose area is the same as a 2D image of the particle.

### Scanning electron microscopy

Prior to SEM, paraformaldehyde fixed samples were pretreated with the following solutions: 50 mM NaN_3_ for 1 h followed by 2% tannic acid for 1 h, 1% osmium tetroxide for 2 h, 1% thiocarbohydrazide for 30 min, and 1% osmium tetroxide overnight. The samples were washed using 10 mM HEPES buffer (pH 7.4) between steps (all Sigma-Aldrich). The samples were then dehydrated in a graded series of aqueous ethanol solutions (50−100%) and oven-dried (2 h at 40 °C) to remove residual moisture. The dried samples were mounted over SEM stubs with double-sided conductivity tape and a thin layer of gold palladium was applied before capturing images using a Quanta 650 SEM (FEI, The Netherlands).

## Additional Information

**How to cite this article**: Bagchi, S. *et al*. Metatranscriptomics reveals the molecular mechanism of large granule formation in granular anammox reactor. *Sci. Rep.* **6**, 28327; doi: 10.1038/srep28327 (2016).

## Supplementary Material

Supplementary Information

Supplementary Video

## Figures and Tables

**Table 1:** Overview of the most important anammox genes detected in the transcriptomes of top and bottom samples.

Annotated Function	Gene	Genbank ID	Protein Length (aa)	Contig Name	Contig Length (nt)	E-value	Identity (%)	Length Mapped (aa)	Percentage Mapped	TPM
Nitrate/Nitrite antiporter	*narK*	kuste2308	390	1847_c0_g2_i1	6255	5E-159	63.75	320	82.05	475.43
	*narK*	kuste2335	406	1847_c0_g2_i1	6255	0	88.43	337	83.00	475.43
Nitrite transporter	*focA*	kusta0004	364	1888_c0_g1_i1	12765	2E-155	81.56	244	67.03	182.81
	*focA*	kustd1720	297	1390_c0_g1_i1	1902	4E-125	68.98	274	92.26	43.02
	*focA*	kustd1721	304	1888_c0_g1_i1	12765	9E-107	63.5	263	86.51	182.81
	*focA*	kuste4324	305	1390_c0_g1_i1	1902	3E-137	71.9	274	89.84	43.02
Nitrite reductase	*nirS*	kuste4136	622	2640_c0_g1_i1	973	1.6	66.67	15	2.41	11.5
	*nirK*	kuste2479	852	1960_c0_g1_i1	14777	0	74.76	515	60.45	80.59
Ammonium transport protein	*amtB*	kustc0381	586	1922_c0_g2_i2	11228	0	78.85	364	62.12	236.67
	*amtB*	kustc1009	520	1778_c0_g1_i1	12225	0	77.2	250	48.08	166.21
	*amtB*	kustc1012	452	1778_c0_g1_i1	12225	3.00E-163	60.95	210	46.46	166.21
	*amtB*	kustc1015	449	2418_c0_g1_i1	2806	6E-88	65.32	124	27.62	119.44
	*amtB*	kuste3690	680	1938_c0_g1_i1	15895	0	60.35	512	75.29	320.25
Hydrazine or hydroxylamine oxidoreductase	*hao*	kusta0043	592	2106_c2_g5_i2	22376	0	90	370	62.50	327.71
	Hao*	kustc0458	555	2094_c0_g14_i8	7233	0	86.24	516	92.97	151.97
	*hao*	kustc0694	657	1955_c1_g1_i2	5836	0	89.19	444	67.60	2944.32
	*hao*	kustc1061	537	1838_c0_g1_i1	2169	0	91.24	388	72.25	3585.92
	*hao*	kustd1340	578	1955_c1_g1_i2	5836	0	87.19	570	98.62	2944.32
	*hao*	kuste2435	500	2147_c1_g11_i3	11047	0	66.74	463	92.60	133.85
	*hao*	kuste2457	434	1915_c2_g3_i6	9020	7E-76	46.48	71	16.36	669.34
	Hao*	kuste4574	585	2112_c3_g3_i6	17457	0	88.96	471	80.51	186.35
Hydrazine Synthesis	*hzsA*	kuste2861	810	2587_c1_g1_i9	6601	0	79.89	363	93.80	902.05
	*hzsB*	kuste2860	354	2154_c1_g5_i8	20691	0	85.8	345	97.46	1303.35
	*hzsC*	kuste2859	387	2154_c1_g5_i5	18812	0	82.62	800	98.77	1303.35
Nitrate reductase	*narG*	kustd1700	1149	1844_c0_g1_i5	12198	0	85.97	720	62.66	746.08
	*narH*	kustd1703	411	2143_c0_g2_i5	31862	0	91.54	260	63.26	468.26
Acetyl-CoA synthetase	*acsA*	kustd1546	654	2117_c0_g1_i2	10448	0	84.89	364	55.66	313.95
	*acsA*	kuste4610	659	1922_c0_g1_i4	3659	3E-88	43.28	134	20.33	554.98
	*acsB*	kustd1545	728	2117_c0_g1_i2	10448	0	87.91	728	100.00	313.95

^*^HAO-like genes kustc0458 and kuste4574 are possible candidates for nitrite reduction in anammox bacteria. aa: amino acids, nt: nucleotides.
