# Supplementary material for: Metatranscriptomics reveals the molecular mechanism of large granule formation in granular anammox reactor
Source: Sci Rep. 2016 Jun 20;6:28327. doi: 10.1038/srep28327 (PMC4913261; doi:10.1038/srep28327)
Supplement: Supplementary Information [file srep28327-s1.pdf]

Supplementary Information

Metatranscriptomics reveals the molecular mechanism of large granule formation in granular anammox reactor

Samik Bagchi[1#], Regina Lamendella[2], Steven Strutt[2], Mark C.M. Van Loosdrecht[3], and Pascal E. Saikaly[1,*]

[1]King Abdullah University of Science and Technology, Biological and Environmental Sciences and Engineering Division, Water Desalination and Reuse Research Center, Thuwal 23955-6900, Saudi Arabia
[2]Juniata College, Biology Department, Huntingdon, PA 16652, USA
[3]Delft University of Technology, Department of Biotechnology, Julianalaan 67, 2628 BC Delft, The Netherlands
[#]Current Address: University of Kansas, Department of Civil, Environmental, and Architectural Engineering, Lawrence, KS, USA 66045

*Corresponding author: Pascal Saikaly (pascal.saikaly@kaust.edu.sa)

Supplementary Methods

Quantitative PCR assays. qPCR was performed on a CFX96 Touch™ Real-Time PCR Detection System (Bio Rad Laboratories Inc., Hercules, CA). All amplifications were performed in triplicate with a reaction volume of 25 µL containing 12.5 µL of iQ SYBR Green Supermix (Bio-Rad, Hercules, CA), 200 nmol/L of each primer and 5 µl of the diluted sample. The annealing temperature of the primers was optimized with temperature-gradient qPCR. Thermal cycling conditions, primer sequences and DNA used as the standards for qPCR calibration are listed in Supplementary Table S1. Two different dilutions (10^{-1} and 10^{-2}) of each sample were amplified to validate the quantification by calculating the difference between cycle threshold (C_t) values for each dilution.

For standard clone preparation, the PCR amplicons were first cloned into a TOPO cloning vector (pCR 2.1-Topo vector, Invitrogen, Carlsbad, CA) according to the manufacturer's protocol. Plasmids from transformed cells were extracted by the PureYield™ Plasmid Miniprep System (Promega, Madison, WI). Copy numbers per microliter were calculated from the concentration of extracted plasmid DNA using the known sequences of the vector and inserts. The accuracy of insert DNA was verified by amplification with gene-specific primers as described in Supplementary Table S1. Standard template DNA was diluted in series and the C_t values for each dilution were plotted against the concentration of each dilution to construct the standard curve. The 16S rRNA gene copy number of anammox, AOB, *Nitrospira* spp. and general bacteria (EUB), and the beta subunit of nitrite oxidoreductase (NxrB) gene copy number of *Nitrobacter* spp. was determined for each sample using the respective standard curves. No-template

controls were included in all qPCR runs. The quantification detection limit for each qPCR assay is provided in Supplementary Table S2.

16S rRNA gene sequencing. Bacterial 16S rRNA genes were amplified using the fusion primer 341F (5'-Lib-L/A-Key-Barcode-CA Linker-CCTACGGGNGGCWGCAG-3') and reverse primer 805R (5'-Lib-L/B-Key-TC linker-GACTACHVGGGTATCTAATCC-3'). The primers used in this study targeted the V3-V4 region of the 16S rRNA gene. A unique 10-bp error-correcting barcode was used to tag each PCR product. PCR was performed using a C1000 Thermal Cycler (Bio-Rad, Hercules, CA) with the following PCR conditions: initial denaturation at 95°C for 5 min, 25 cycles of denaturation at 95°C for 30 s, annealing at 55°C for 30 s, and extension at 72°C for 30 s, and a final extension at 72°C for 5 min. Triplicate PCR products from each sample were pooled and confirmed by gel electrophoresis and then purified using the Qiaquick gel extraction kit (QIAGEN, Valencia, CA) according to the manufacturer's protocol. The concentration of the PCR products was measured on a Qubit® 2.0 Fluorometer using the PicoGreen® dsDNA quantitation assay (Invitrogen, Carlsbad, CA). The purified barcoded amplicons from each sample were then pooled in equimolar concentrations and sequenced on the Ion Torrent PGM genome sequencer using 318 chips (Life Technologies, Carlsbad, CA), according to the manufacturer's instructions. A total of 5,260,877 reads was generated. The sequencing reads were deposited at NCBI Sequence Read Archive (SRA) under project accession number SRX745120.

Processing of sequencing data. After sequencing, the individual sequence reads were filtered within the PGM software to remove low-quality and polyclonal sequences. All PGM quality filtered data were exported as FastQ files, split into constituent *.fasta and *.qual files and subsequently processed using the Quantitative Insights Into Microbial Ecology (QIIME v1.9.0) pipeline[1]. Raw reads were first trimmed to remove low quality ends from reads using quality trimming in cutadapt v 1.9.1[2]. The end trimmed sequences were demultiplexed to trim the barcodes and primers and then low-quality sequence reads outside the bounds of 200 and 600 bp, all sequences containing ambiguous bases, sequences with 6 homopolymers and sequences with quality scores less than 25 were removed. Sequences were clustered into operational taxonomic units (OTUs) using uclust[3] with a 97% sequence identity threshold. Representative sequences from each OTU were aligned using PyNAST[4] and the taxonomic identity was assigned by the Greengenes database (release 13.8) using the open reference OTU picking workflow in QIIME. Singletons were removed from the sequences as they can be potential artifacts and our target was the dominant bacterial population. Chimeric sequences were identified and removed from the aligned sequences using Chimera Slayer as implemented in QIIME. To compare all samples at equal sequencing depth, samples were normalized to the minimum number of sequences (i.e., 10,186 reads/sample) by random sampling (without replacement). Further analysis was performed to detect outliers among samples, which were removed from the 16S rRNA dataset using the outlier detection algorithm in PC-ORD with a cutoff of three standard deviations[5]. A total of 71,302 reads encompassing 4,664 OTUs passed the QA/QC steps.

To estimate sampling completeness and calculate the probability that randomly selected amplicons from a sample had already been sequenced, Good's coverage and rarefaction analysis was performed on the resulting OTU table. The community composition was plotted using summarize_taxa_through_plots.py in QIIME.

Table S1 Read numbers and statistics of metatranscriptomic data obtained from granular anammox reactor.

	**D26_top**	**D26_btm**	**D52_top**	**D72_top**	**D72_btm**
Total Reads	18880885	17202635	24287121	17971589	17237179
Normalized Reads	2355887	1977227	1935793	2499225	2040876
Assembled Contigs	12052	9375	7869	9815	11082
Average contig length (nt)	937	984	973	952	764
nt total	5859	4641	4859	5510	5951
nt unique	1951	1731	1812	1892	2056
COG total	5374	4336	3201	4217	4483
COG unique	1407	1242	1102	1227	1300
% COG unique	26.18	28.64	34.43	29.10	29.00
KEGG total	8106	6353	5262	6501	7135
KEGG unique	1636	1402	1248	1404	1528
% KEGG unique	20.18	22.07	23.72	21.60	21.42
Mapping of raw reads to anammox genome					
Coverage (% sequence mapped)	6.48	6.27	6.01	6.41	5.95
Bases mapped	8870952	7914681	13194632	6943775	6424144
Reads mapped	273329	264657	253446	270217	250918

Table S2 Primers, thermal cycling conditions and standards used for qPCR

Target genes	Primers	Nucleotide sequence (5′–3′)	Thermal cycling	Standard	Detection Limit (copies/mL of biomass)	Reference
16S rRNA gene of anammox bacteria	Amx368f<br>Amx820r	TTCGCAATGCCCGAAAGG<br>AAAACCCCTCTACTTAGTGCCC	94°C-10min, 30 × (94°C-30s, 55°C-30s, 72°C-1min), 72°C-7 min	Anammox biomass	1.8×10^3	6
16S rRNA gene of AOB	CTO189f A/B<br>CTO189f C<br>CTO654R	GGAGRAAAGCAGGGGATCG<br>GGAGGAAAGTAGGGGATCG<br>CTAGCYTTGTAGTTTCAAACGC	94°C-10min, 30 × (92°C-1min, 57°C-1min, 68°C-2 min)	Anammox biomass	2.1×10^2	7
NxrB[a] gene of *Nitrobacter* spp.	NxrB 706F<br>1431R	AAGACCTAYTTCAACTGGTC<br>CGCTCCATCGGYGGAACMAC	95°C-5min, 40 × (95°C-40s, 56°C-30s, 72°C-30s), 72°C-10min	Nitrobacter sp[b]	1.0×10^1	8, 9
16S rRNA gene of *Nitrospira* spp.	Nspra 675f<br>746r	GCGGTGAAATGCGTAGAKATCG<br>TCAGCGTCAGRWAYGTTCCAGAG	95°C-10min, 40 × (95°C-20s, 58°C-1min, 72°C-40 s)	Anammox biomass	1.0×10^1	10
16S rRNA gene of universal bacteria	338F<br>518R<br>P338-IIf	ACTCCTACGGGAGGCAGCAG<br>ATTACCGCGGCTGCTGG<br>ACACCTACGGGTGGCTGC	96°C-3min 40 × (95 °C-30s, 55 °C- 30s, 72°C -30s), 72°C-5min	Anammox biomass	2.9×10^2	11,12

[a]Gene encoding the beta subunit of nitrite oxidoreductase
[b]*Nitrobacter* sp. enrichment culture provided by Dr. Eva Spieck at the University of Hamburg

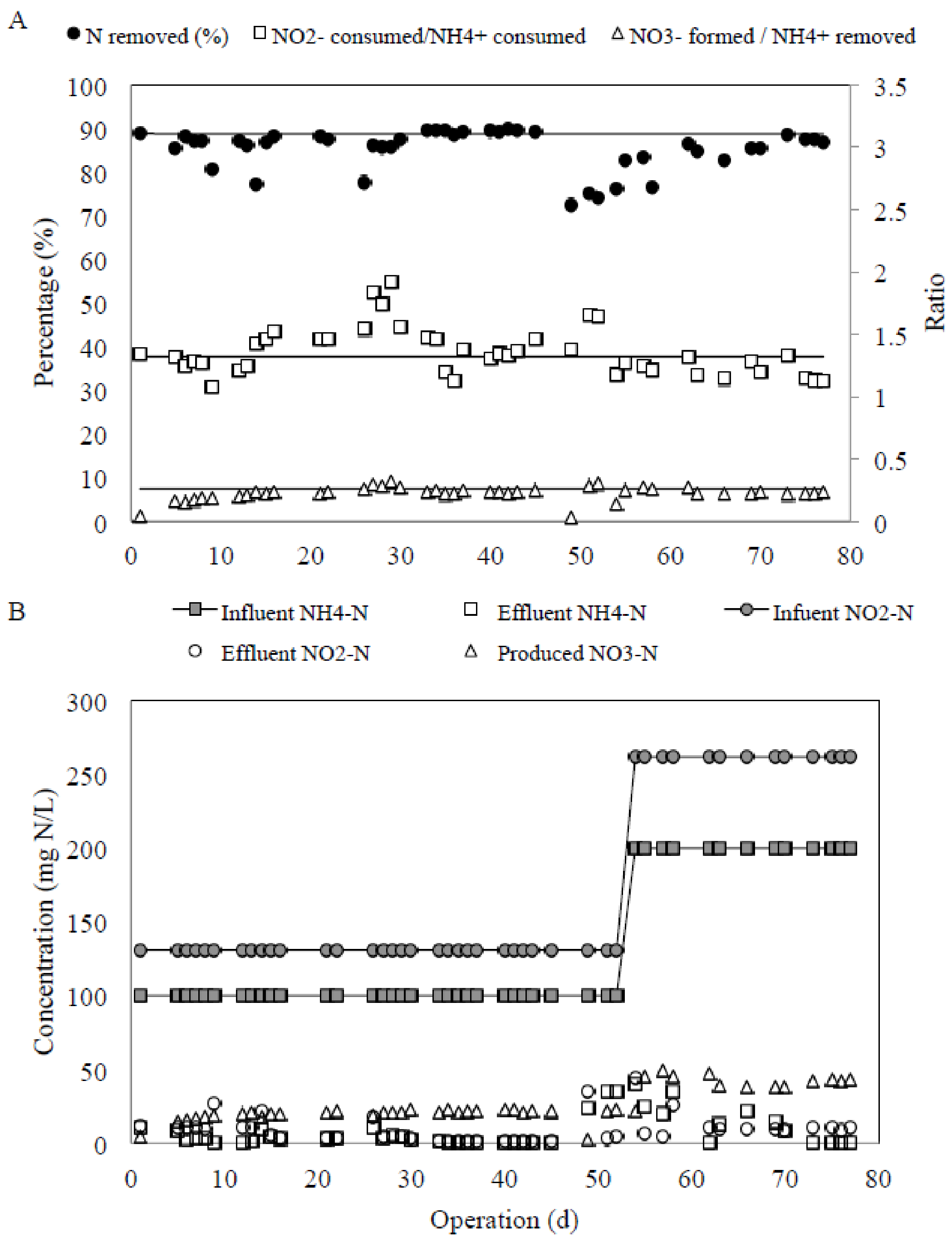

Figure S2 – Performance of Anammox SBR showing the removal efficiency and consumed nitrite to ammonia ratio and nitrate formation ratio. Horizontal lines show the optimal conditions (A). Influent and effluent nitrogen concentrations showing performance of SBR (B).

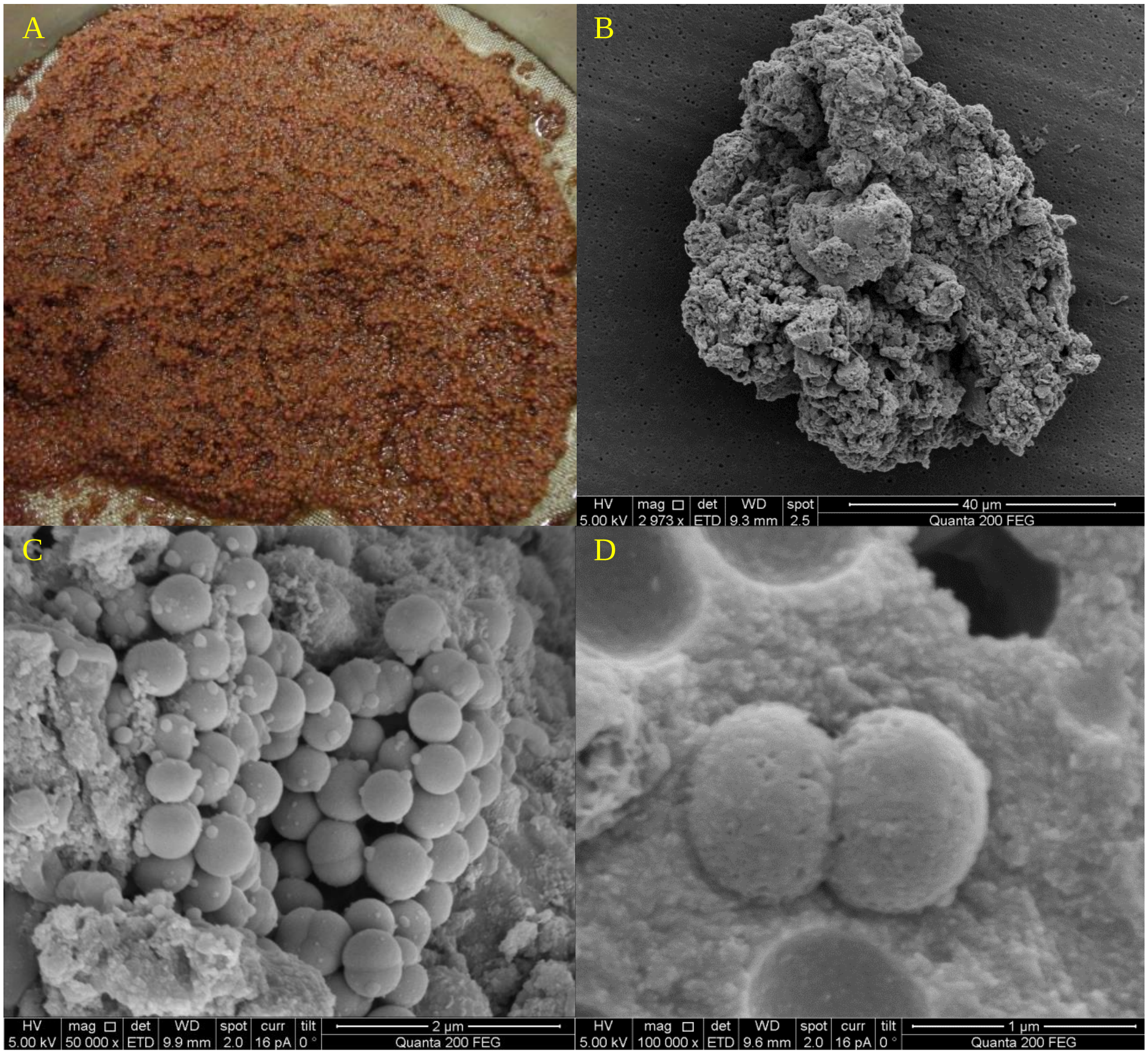

Figure S2 Anammox granules (A). SEM images of anammox granules (B) with the localization of anammox cell-like clusters inside the granules (C) and dividing anammox like coccoid cells (D). The magnification and scale-bar is shown at the bottom of the SEM. Figure S2A was taken by Samik Bagchi.

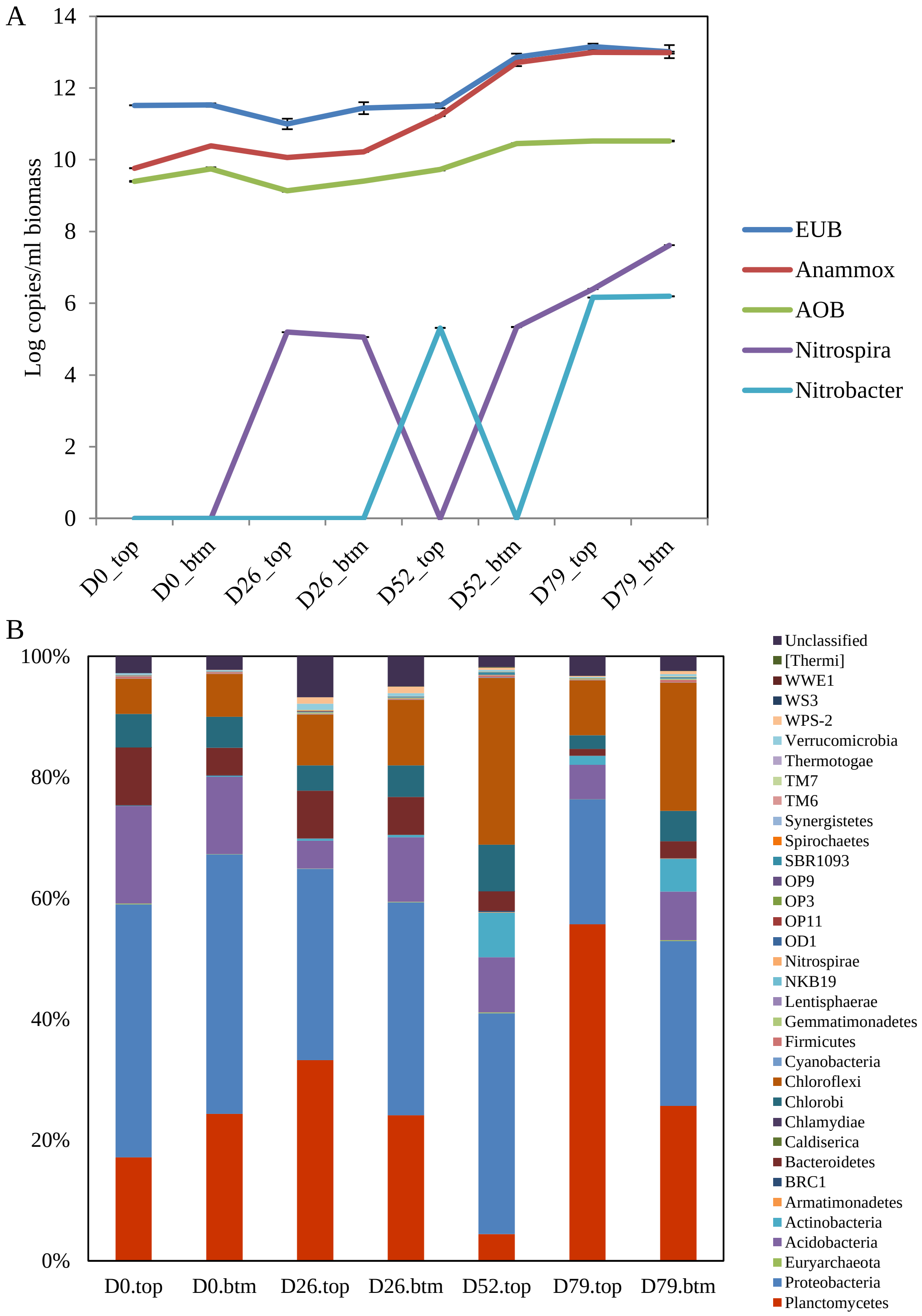

Figure S3 Temporal variation of gene copy number of major nitrifying bacteria as measured by qPCR. The error bar indicates the average of triplicate samples (A) and the relative abundance of all phyla in Anammox SBR as measured by amplicon 16S rRNA gene sequencing. 'unclassified' are sequences not matched to any know organisms (B).

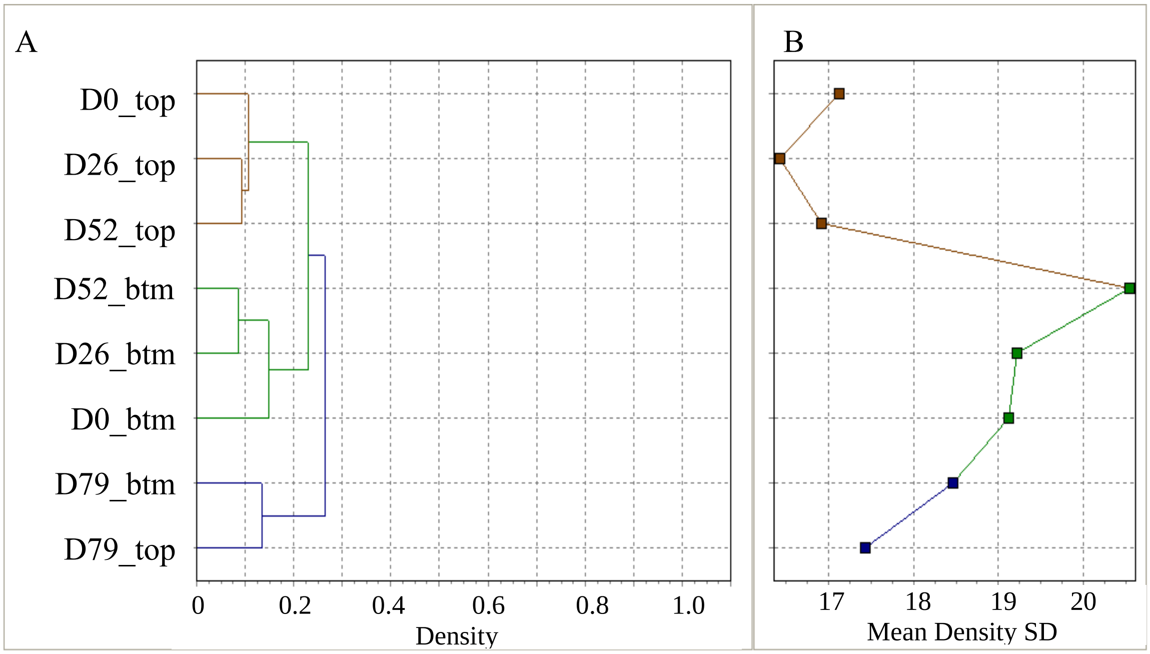

Figure S4 Dendrogram clustering the top and bottom biomass based on density (A) and the overall mean density trend plot of the respective samples (B). Density was measured as the mean intensity SD, which is the average of the pixel greyscale levels in the object.

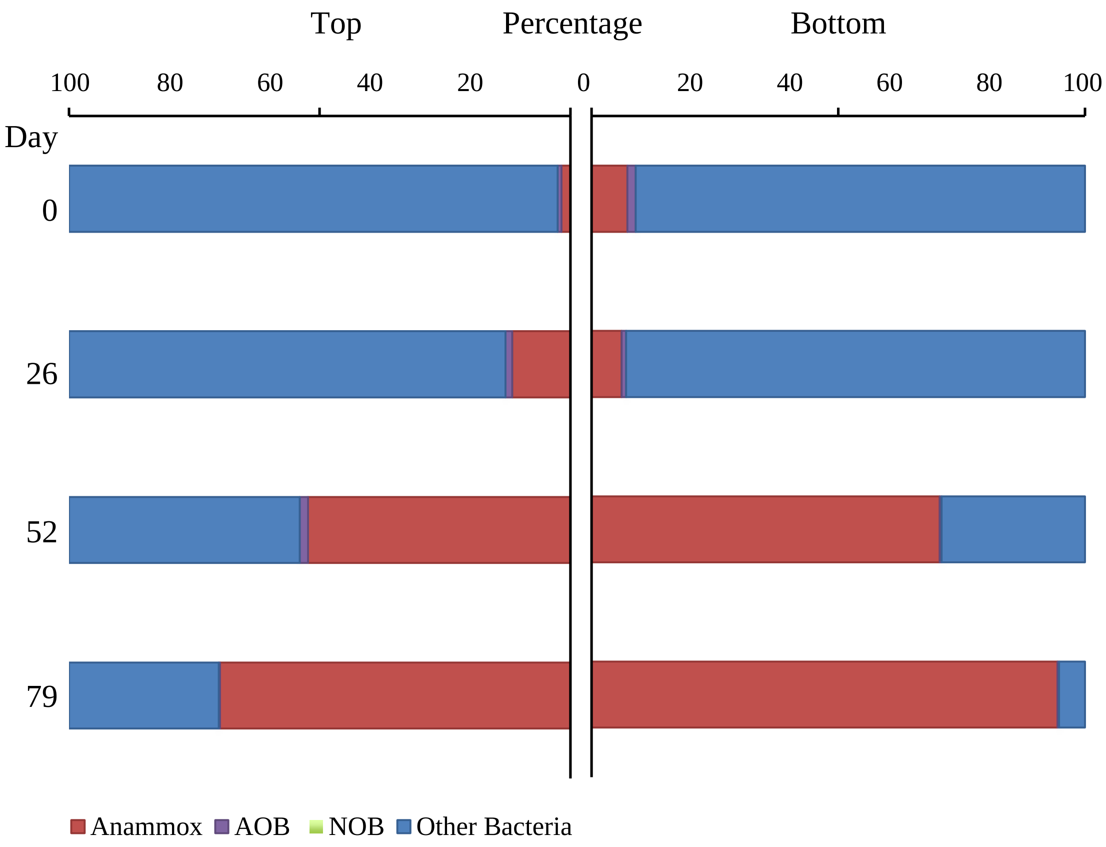

Figure S5 Comparison of the relative abundance of major N-cycle microorganisms between the top biomass (left) and the bottom biomass (right) based on qPCR.

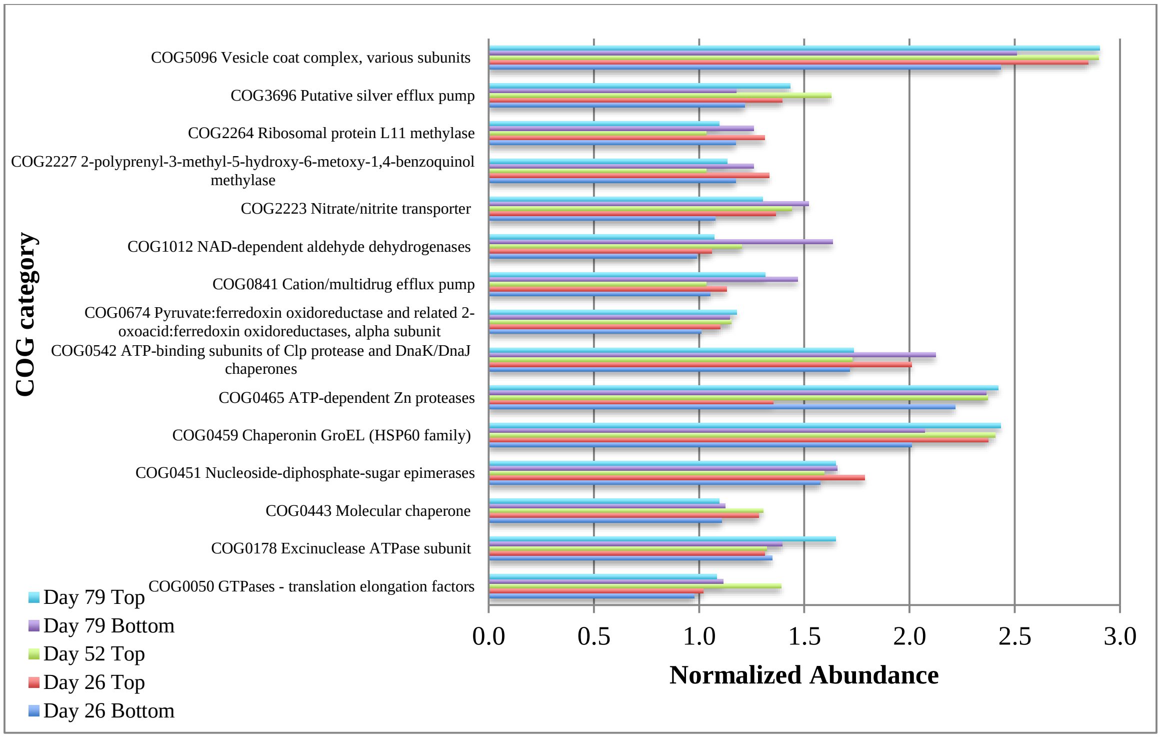

Figure S6 Normalized relative abundances of the top 15 most abundant COGs. The abundance of metatranscriptomic reads assigned to a given COG was normalized to the number of rpoB reads. The top 15 most abundant COGs were selected based on their average normalized abundance across all five samples. Additionally, each of the top 15 COGs had to be present in all samples.

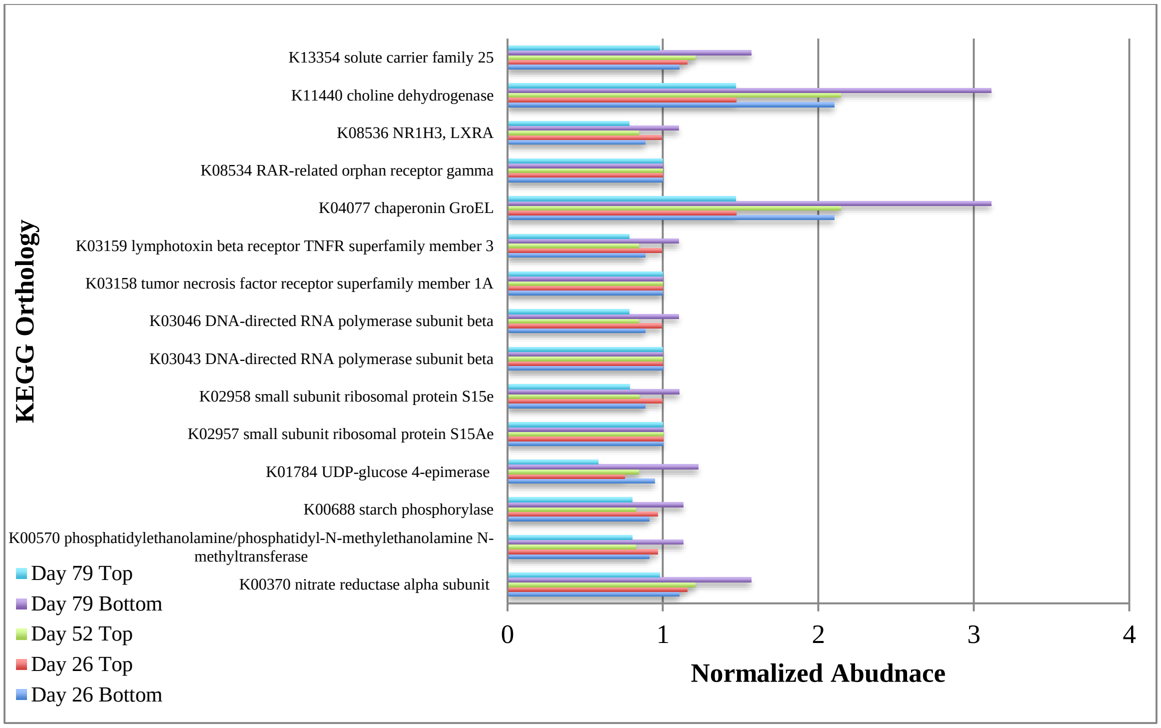

Figure S7 Normalized relative abundances of the top 15 most abundant KEGG orthologs. The abundance of metatranscriptomic reads assigned to a given KEGG ortholog was normalized to the number of rpoB reads. The top 15 most abundant KEGG orthologs were selected based on their average normalized abundance across all five samples. Additionally, each of the top 15 KEGG orthologa had to be present in all samples.

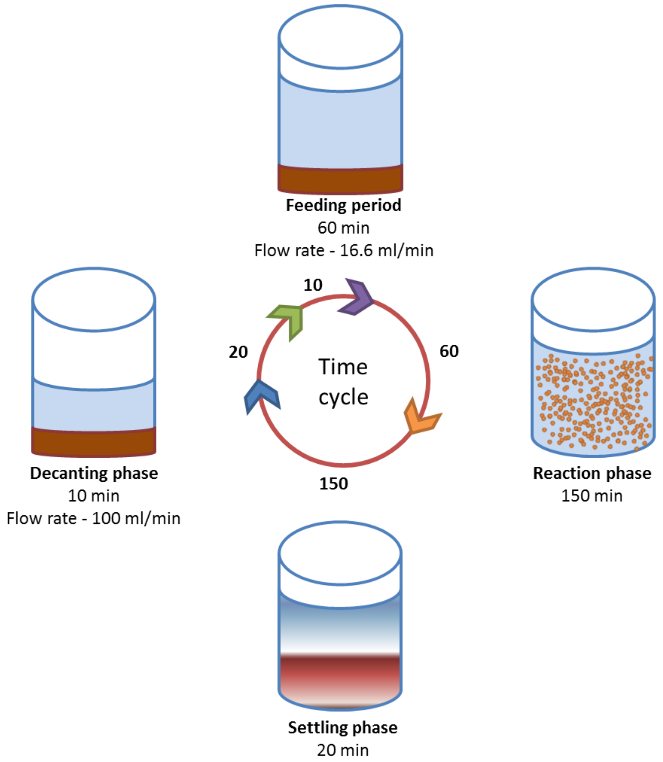

Figure S8 A schematic of the operation stages in the laboratory SBR consisting of feeding, reaction period with mixing with 5% CO_2 + 95% Argon gas, settling period and decanting period. Figure S8 was created by Samik Bagchi.

Supplementary Animation 1 Animation showing proposed molecular mechanism of large granule formation. Supplementary Animation 1 was created by Academic Writing Services, KAUST, Saudi Arabia.

References

1. Caporaso JG*, et al.* QIIME allows analysis of high-throughput community sequencing data. *Nat Meth* **7**, 335-336 (2010).

2. Martin M. Cutadapt removes adapter sequences from high-throughput sequencing reads. *EMBnet journal* **17**, pp. 10-12 (2011).

3. Edgar RC. Search and clustering orders of magnitude faster than BLAST. *Bioinformatics* **26**, 2460-2461 (2010).

4. Caporaso JG, Bittinger K, Bushman FD, DeSantis TZ, Andersen GL, Knight R. PyNAST: a flexible tool for aligning sequences to a template alignment. *Bioinformatics* **26**, 266-267 (2010).

5. McCune B, Grace JB, Urban DL. *Analysis of ecological communities*. MjM software design Gleneden Beach, OR (2002).

6. Schmid MC*, et al.* Biomarkers for in situ detection of anaerobic ammonium-oxidizing (anammox) bacteria. *Applied and environmental microbiology* **71**, 1677-1684 (2005).

7. Geets J*, et al.* Real-time PCR assay for the simultaneous quantification of nitrifying and denitrifying bacteria in activated sludge. *Applied microbiology and biotechnology* **75**, 211-221 (2007).

8. Winkler MK, Kleerebezem R, Khunjar WO, de Bruin B, van Loosdrecht MC. Evaluating the solid retention time of bacteria in flocculent and granular sludge. *Water research* **46**, 4973-4980 (2012).

9. Koch, H. Ecophysiological investigation of nitrite-oxidizing bacteria of the genus Nitrospira, Thesis, Unversity of Vienna (2009).

10. Graham DW, Knapp CW, Van Vleck ES, Bloor K, Lane TB, Graham CE. Experimental demonstration of chaotic instability in biological nitrification. *The ISME journal* **1**, 385-393 (2007).

11. Marchesi JR*, et al.* Design and Evaluation of Useful Bacterium-Specific PCR Primers That Amplify Genes Coding for Bacterial 16S rRNA. *Appl Environ Microbiol* **64**, 795-799 (1998).

12. Muyzer G, De Waal EC, Uitterlinden AG. Profiling of complex microbial populations by denaturing gradient gel electrophoresis analysis of polymerase chain reaction-amplified genes coding for 16S rRNA. *Applied and environmental microbiology* **59**, 695-700 (1993).
